# Consultations between Immigrant Patients, Their Interpreters, and Their General Practitioners: Are They Real Meetings or Just Encounters? A Qualitative Study in Primary Health Care

**DOI:** 10.1155/2013/794937

**Published:** 2013-02-05

**Authors:** Eivor Wiking, Jan Sundquist, Nouha Saleh-Stattin

**Affiliations:** ^1^Centre for Family Medicine (CeFAM), Department of Neurobiology, Caring Sciences and Society, Karolinska Institutet, Alfred Nobels Allé 12, 141 83 Huddinge, Sweden; ^2^Department of Clinical Sciences, Center for Primary Health Care Research, Malmö, Lund University/Region Skåne, 221 00 Lund, Sweden; ^3^Stanford Prevention Research Center, Stanford University, Stanford, CA 94305-5411, USA

## Abstract

*Objective*. In Sweden, about 19% of residents have a foreign background. Previous studies reported immigrant patients experience communication difficulties despite the presence of interpreters during consultations. The objective of this study was to gain insights into the participants' perceptions and reflections of the triangular meeting by means of in-depth interviews with immigrant patients, interpreters, and general practitioners (GPs). *Method*. A total of 29 participants—10 patients, 9 interpreters, and 10 GPs—participated in face-to-face interviews. Content analysis was used to process the interview material. *Results*. Six themes were generated and arranged under two subject areas: the interpretation process (the means of interpreting and means of informing) and the meeting itself (individual tailored approaches, consultation time, the patient's feelings, and the role of family members). *Conclusion*. This paper highlights feelings including frustration and insecurity when interpretation and relationships are suboptimal. Strategies for immigrant patients, interpreters, and GPs for getting a successful consultation may be needed. To transform the triangular meeting from an encounter to a real meeting, our results indicate a need for professional interpreters, for GPs to use a patient-tailored approach, and sufficient consultation time. *Practice Implications*. Use of professional interpreters is recommended, as is developing cultural competence.

## 1. Introduction

All patients have the right to equal access to health care that meets their individual needs. The Swedish Health and Medical Services Act (1982:763) states that the objective of health care is good health and care on equal terms for the whole population. Section  2 (b) states that the patient should be given individually tailored information on 1. His state of health 2. The methods of examination, care, and treatment that exist.

Between the end of the Second World War and the 1960s, Sweden had a large influx of labour immigrants. Since the 1980s, immigration has been dominated by refugees and/or individuals with family members in Sweden. Sweden is a multicultural society, with 19% of its population in 2011 having a foreign background [1].

An assessment of primary care services conducted in London by Campbell et al. [2] showed that younger patients and nonwhite ethnic minority patients rated the care they received less positively than older patients and ethnic majority patients. A Swedish study by Hjern et al. [3] found that immigrants from Chile, Turkey, and Iran have a less satisfactory self-reported health status and attended more consultations than Swedish-born residents. Van Wieringen et al. [4] showed that mutual understanding is poorer in consultations with foreign-born patients compared to those with native-born patients. When 80 general practitioners (GPs) in New Zealand were interviewed about the use of non-English languages in general practice and its effect on the quality of the consultation [5], the majority (73 out of 80) experienced language difficulties which influenced the encounter. 

Five key predictors of culture-related communication problems in medical consultations were identified in a review by Schouten and Meeuwesen [6]: (1) cultural differences in explanatory models of health and illness; (2) differences in cultural values; (3) cultural differences in patients' preferences for doctor-patient relationships; (4) racism/perceived biases; and (5) linguistic barriers. Culture was found to be only one dimension in consultations where communication was difficult. In a study by Wachtler et al. [7] it was shown that the GP's focus was more on the individual rather than on the individual's culture.

Conversation analytic studies have illuminated interactions between healthcare professionals and patients. Pilnick et al. [8] have highlighted issues concerning (1) practical problems and different dilemmas that arise in practitioner-patient interaction, (2) interaction between healthcare practitioners, and (3) new technologies and healthcare interaction. Physicians received different conversational clues from ethnic minority patients compared to Dutch patients in cases of poor mutual understanding in a study by Meeuwesen et al. [9]. The more affectively GPs behave (concerning social behaviour, agreement, paraphrasing, showing concern, reassurance, reflection, and disagreement) during the consultation, the more questions patients ask, which influences patient participation and satisfaction, as shown in a study from The Netherlands [10]. 

Using a questionnaire about triangular meetings between immigrant patients, interpreters, and GPs in primary health care in Stockholm, we previously found that 63% of patients were satisfied with their consultations [11]. However, 50% of patients reported communication difficulties, despite the presence of an interpreter. A patient-centred strategy, professional interpreters, and cultural awareness were emphasised for achieving good communication and hence successful consultations.

In order to complement the existing knowledge and possible theories about reasons for communication problems in the triangular meeting, we decided to explore more deeply what happens during the meeting between the three participants. A *meeting* is an assembly of people for a particular purpose [12]. An *encounter* is an unexpected or casual meeting with someone [12]. A *meeting* is more of an in-depth experience whereas an *encounter* is more superficial.

The objectives of this study were to gain insights into participants' perceptions of and reflections on consultations by means of in-depth interviews with immigrant patients, interpreters, and general practitioners and to perform an in-depth qualitative analysis of the interviews.

## 2. Method

### 2.1. Participants

Participants in the following three categories were selected on the basis of participation in the same consultation: immigrant patients from Chile, Turkey, and Iran, their interpreters, and GPs at five different primary health care centres (PHCCs) in Stockholm. The interviews took place between 2004 and 2010 one-two weeks after the consultation. The participants received both verbal and written information and provided informed consent. Professional interpreters who were not included in this study were booked for the patients' interviews. A total of 29 individuals—10 patients, 9 interpreters, and 10 GPs—participated in a total of 30 face-to-face interviews. One interpreter was interviewed twice. Because the content of the two interviews with this interpreter differed, both were included in the analysis. Seven GPs were from Sweden and the rest were born abroad. All GPs had a lot of experience of working in PHCCs with high proportions of immigrant patients. Three of the interpreters spoke Spanish as their mother tongue, three Persian, and three Turkish. All had worked as interpreters for many years—in six cases more than 10 years—except for one, who had only worked for 2 months. Seven of the interpreters were authorised (public authority exercised by “Kammarkollegiet,” Legal Financial and Administrative Services Agency). The participants' characteristics are shown in Table 1. 

### 2.2. Interviews

In-depth interviews took place at five different PHCCs in Stockholm County and lasted 45–60 minutes. The interviews were led by one of the researchers (EW) using an interview guide (see Appendix). Professional interpreters who were not included in this study were booked for the patient interviews. They started with open questions encouraging the participants to elaborate on their experiences and reflections. The interviews were tape-recorded and transcribed verbatim. Each participant was sent a written copy that had been translated into his/her native language by a professional interpreter. The participants were asked to comment on and confirm the accuracy of the content and to return their copies to the researcher (EW). Three patients returned their copies, one with comments in his own language, which were subsequently translated into Swedish. Six of the interpreters returned their copies, three with comments. Six of the GPs returned their copies, three with comments. The comments were reviewed, but did not contribute any additional information to the interview content.

### 2.3. Analyses

Two of the researchers (EW and NSS) used content analysis [13] to independently analyse the interviews for each group of informants separately before comparing and discussing their results. 

In content analysis, themes are created by condensation. Condensation involves summarising what appears in the text using a description that is as similar to the text as possible. Next, an interpretation of the underlying meaning of the condensed text is provided. Finally, the underlying meanings are linked together as subthemes and themes. An example of the analysis process is shown in Table 2.

## 3. Results

Our analysis generated six themes common to the three groups (patients, interpreters, and GPs), which we categorised into two subject areas: the interpretation process (*the means of interpreting and means of informing*) and the meeting itself (*individual tailored approaches, consultation time, the patient's feelings regarding interpretation, and the role of family members*). We will use citations from the interviews to illustrate the themes using the following abbreviations: P = patient, IP = interpreter, and GP = general practitioner. 

### 3.1. Patients' Perspectives

#### 3.1.1. Interpretation Process

The interpretation process consisted of two different components: linguistic and cultural interpretations. Linguistic interpretation includes translation between two languages, as well as explicit explanation of medical terms, for example “migraine.” In cultural interpretation, the patient's cultural perspective is taken into account. The interpreter's personality and own strategies appeared to be important factors in the interpretation process in the means of interpreting and means of informing.

*Means of Interpreting.* The means of interpreting can be defined as either the mere translation or communicating patients' wishes and feelings, with or without body language. 

The majority of the patients felt that having a professional interpreter was important for a good patient-GP relationship, with the interpreter's role being to establish trust and create a good atmosphere. It was important for the patient to feel free to express exactly what he or she wanted to say.

*P10: “I want the interpreter to translate or convey exactly how I feel, in other words, my feelings, emotions, and experiences, that is, not only the verbal thing.”*

*Means of Informing*. The means of informing include the importance of professional interpretation in both directions between the patient and the GP. 

Some patients expressed the importance of adjusting information to their culture and level of knowledge about body functions. They also stressed the importance of translating medical terms into everyday language.

*P3: “… there are medical terms used in medicine. There are also English medical terms and then even I have difficulties to understand these terms. I speak everyday language just as we do you and me.”*

#### 3.1.2. The meeting

According to the participants, a successful meeting between the patient and the GP during the consultation requires adapting to the individual patient. Other important factors were consultation time, the patient's feelings, and the role of family members. 

*Individual Tailored Approaches. *Some patients felt that a kind response from the GP, with polite and respectful treatment and a focus on the patient as a whole person was important.

*P7: “The doctor is very kind when she speaks to me, examines well, at least cares for me … very good doctor. She asks me questions about my family members, if they had diseases.”*

*Consultation Time.* The time element is crucial for fulfilling the three participants' expectations and needs of the consultation. Some patients commented that the consultation time was insufficient. The patient has a need to tell the GP everything at the first visit, to be properly examined, and to be able to ask questions.

*P5: “We would like to talk about all our problems during the first visit. One is sad when they say that the time is up. It is like being half-examined.”*

*The Patient's Feelings Regarding Interpretation.* The patient's and GP's dependence on the interpreter may give a feeling of uncertainty about whether the information is interpreted correctly. 

A feeling of frustration was expressed by the majority of the patients. One of them said.

*P6: “One feels frustrated when one does not understand everything.”*

*The Role of Family Members.* Family members assume different roles in the consultation: taking over the interpreter's role, checking the interpretation, and supporting the patient. Some of the patients commented that the interpreter's role is sometimes assumed by a family member who speaks Swedish, providing the patient with a sense of security. Another aspect of this role is to support the patient in their treatment or to act as an interpreter in acute medical situations.

*P5: “… when we make an appointment in an acute situation and no interpreter is available … then either my daughter or someone else from the family who knows Swedish accompanies me and helps to interpret …”*

### 3.2. Interpreters' Perspectives

#### 3.2.1. Interpretation Process

*Means of Interpreting.* The majority of the interpreters described their different ways of interpreting. It was important to translate every word precisely; to be neutral not to disrupt the dialogue between the patient and the GP. Others described the difficulties when the GP did not know how to work with an interpreter.

*IP10: “… sometimes it is more difficult when the care provider is not familiar with this technique. Either he talks too much or questions what has been said the whole time, before you have had time to interpret. One is interrupted all the time.”*

*Means of Informing*. Different cultures have different rules about telling patients the truth about their illnesses and prognoses. Often the explanation behind not telling patients is a wish to support them. Sometimes family members are more informed than the patient, which might be an ethical dilemma for the GP.

*IP8: “Let's say that it is an elderly patient. The GP can tell the children then … maybe in some smooth way, eventually tell the patient or not, or tell a little …”*

Two interpreters commented that the way to provide information to the patient must be adapted to the patient's level of knowledge about the body, even for written information. This is the responsibility of the interpreter, the GP, or collaboration between them.

*IP2: “… Sometimes I have to say it: he is uneducated or she is educated …”*

*IP5: “… Perhaps written information would be easier, since everyone will get the same information. They have the right to read it.”*

#### 3.2.2. The Meeting

*Individual Tailored Approaches.* The majority of the interpreters pointed out that the GP's approach to the patient ought to be more individual, including listening, patience, respect, and responding to the patient's needs and wishes. In some cultures, elderly patients perceive the GP as a “medical authority” who makes the decisions.

*IP3: “… Especially elderly patients … they do not want to decide themselves. They want to have a medical authority.”*

One interpreter expressed the need to intervene when the GP is unaware of the patient's dissatisfaction or fear.

*IP6: “… If the patient is scared about a reply, if I am a good interpreter, I at least try to get the GP to understand that he/she is dealing with a patient who is ill, or believes that he/she is, and perhaps needs more information or reassurance ….”*

*Consultation Time*. The majority of the interpreters felt that the consultation time was never sufficient. They experienced frustration since establishing a relationship with the patient when the GP is stressed will be difficult to accomplish. The patient's need to present his or her story is important to respect. One interpreter commented that if the GP is running late it delays her as well, meaning that she will not be able to do her job properly.

*IP6: “… Even if the doctor is under stress and does not want to hear everything the patient says in detail, or is not listening the whole time, the interpreter has to do what is needed. The brain is not a factory for transforming words from one language to another. One needs a lot of imagination and undivided presence to convey the intended message in the best way.”*

*The Patient's Feelings Regarding Interpretation. *It happens that the patient asks the interpreter how long they have been in Sweden. To have a third person present during the consultation with the GP may be stressful to the patient and could cause uncertainty.

*IP6: “… Many times they ask ‘How long have you been here?' to check whether my language ability is good enough … they are stressed, they are uncertain … They must give space to another person suddenly … in their relationship with the GP …”*

*The Role of Family Members. *Family members may interfere by interpreting incorrectly or providing incomplete information. Delivering a cancer diagnosis is an example of a situation where GPs in Sweden must inform the patient, but the family members may choose not to tell the patient the whole truth. One interpreter expressed it as follows.

*IP2: “… In Sweden, the doctor tells the patient directly that he has got cancer. But we do not do that in our countries. It is a difficult situation for an interpreter. We have to tell the patient what the GP says … one tells the family members and they explain gradually for the patient. Especially elderly patients … they may lose the desire to live and do not struggle anymore.”*

### 3.3. GPs' Perspectives

#### 3.3.1. Interpretation Process

*Means of Interpreting. *One GP described the interpreter as just a voice in the room. During the consultation, the GP has to think constantly how to formulate questions and provide information through the interpreter. Some GPs observed that different interpreters use different techniques, with some of them being empathetic and even translating the patient's feelings.

*GP3: “Some interpreters are just like walls … some sort of language machines with no facial expression, no eye contact with either the patient or me as a doctor. Others are more empathetic …”*

*Means of Informing*. Both interpreters and GPs commented that the way to provide information to the patient must be adapted to the patient's level of knowledge about the human body.

*GP5: “… you have to adjust your way of communicating … where the differences in education levels, or what part of the original country the patient comes from, has greater significance than the country they come from.”*

#### 3.3.2. The meeting

*Individual Tailored Approaches.* The GPs felt it was important to have a mutual understanding with the patient, to see them as an individual, and to listen and try to determine the main reason for the consultation.

*GP5: “… with each individual, I have to listen and try to figure out what it is all about.”*

One GP commented that it is important to be professional, to show respect and understanding for the patient's earlier experiences and autonomy, which sometimes may lead to compromises. Another GP commented that it is important in every consultation to show respect for the patient without any prejudices about their background.

*GP4: “… most important in providing care for the patient is not having prejudices. To see the patient you have in front of you as a human being, regardless of where they come from … You must meet each individual with respect.” *

Another GP commented that even when the patient has no or little command of Swedish, direct communication improves contact, making consultations more active and revealing.

*GP8: “… I think I can get so much more from a visit when I speak to the patient … I have women who speak a little Swedish … when the interpreter fails to show up … the visits are more vigorous … better contact with the patient.”*

*Consultation Time*. More than half of the GPs felt that it was desirable to have longer consultation times because the interpretation takes time. Another time-consuming factor is adjusting information to match the patient's level of knowledge about the body and obtaining knowledge about the association between symptoms and psychosocial factors.

*GP7: “We do not have longer consultations when an interpreter is present. There is also another problem due to the patient's lack of knowledge about how the body works, which may give you an inadequate medical history … It can take an incredibly long time to find out what it is about …”*

*The Patient's Feelings Regarding Interpretation. *Half of the GPs described patients' expectation to be provided with professional interpretation with no judgment.

*GP7: “…it happens often that we are talking past each other and that the patient feels offended by something that they feel that I have said.” *

*The Role of Family Members*. All GPs commented on the roles of family members. Uncertainty as to whether the interpreter may divulge confidential information may result in patients refusing to have an interpreter.

*GP10: “… the patients do not always have confidence in the interpreter's translation. In that case, it may be nice to have a family member in the situation with them.”*

The participants' different perspectives concerning the themes are illustrated in Table 3.

Variation in the quality of the interpretation and the relationship between the patient and the GP could affect the outcome, as illustrated in Table 4. 

## 4. Discussion and Conclusion

### 4.1. Discussion

In this study, our main findings have indicated that the dynamics between the three participants during a consultation influence the relationship between the GP and the patient and therefore also mutual understanding. This is in congruence with a study by van Wieringen et al. [4], who demonstrated that for a successful consultation and to satisfy the different persons involved, it is important that both the meeting itself and the interpretation process are satisfactory. 

Even though the three groups of participants had themes in common, they sometimes had different perspectives on these themes. Concerning means of interpreting, patients and GPs had the same or similar perspectives. All three groups of informants had similar perspectives on the means of informing. Also, when looking at the “individual tailored approach,” we found similar perspectives, except for the interpreters, who stressed that “medical authority” was important for some patients. All three groups commented that establishing good contact and a good relationship, as well as exchanging information between the patient and his or her GP through an interpreter, is often time consuming. Most interpreters commented that patients complained about consultation times. The patients expressed a sense of frustration during the consultation, while interpreters expressed stress and uncertainty. The GPs reported that some patients had felt offended about what had been said and pointed out patients' expectations of having a professional interpreter without any judging. Patients and GPs see positive sides to the role of family members, whereas interpreters see it as a threat and as negative. GPs have both positive and negative views about it. 

Baker et al. [14] found that patient satisfaction is related to possibilities to communicate through an interpreter. Patients have the right to decide if they want to have an interpreter present or not. At the same time, the GP may ask for one if the communication does not work, in agreement with our results. The interpretation must be correct in both directions to avoid misunderstanding, which may lead to incorrect assessment and treatment by the physician. To use an interpreter may be frustrating for the patient, since they do not have the possibility to check the interpretation.

The meaning of “professional interpreters” varies from one country to another. This variation has implications for everyday encounters for which interpreters are booked. Different countries have different skill requirements for examinations and certifications administered by a knowledgeable authority (The Legal, Financial and Administrative Services Agency in Sweden). 

In our study some patients wanted their feelings to be interpreted, but for a professional well-trained interpreter who has been taught not to interpret anything other than what is said, it may be against his/her ethics to interpret unsaid emotions. Sometimes the patient feels uncertain about the interpreter's professional confidentiality, despite information about it. It is important for the patient's feelings of trust and confidentiality to listen to the patients' wishes concerning the interpreter's behaviour during communication, including respect and a professional neutral attitude [15]. More interpreter errors of clinical significance occur when “untrained ad hoc interpreters” are used [16]. Our results indicated that family members, who know the patient well, might be able to provide valuable additional information that could facilitate the consultation and help the GP to establish a relationship with the whole family, thus valuing family members' engagement and language skills as important resources [17]. They may take the role of a regulator to ensure correct interpretation when the patient is uncertain about the interpretation quality. It may also be a risk to use a family member as an interpreter, since he may give an incorrect or inadequate interpretation of the medical history [18]. The GP may be uncertain as to whether information has been lost and mistakes have been made. Rosenberg et al. [17] showed that family interpreters may play care-giving roles with their own agendas, that GPs treat them as caregivers and partners, and that they may not act according to official rules for interpretation. It is important to inform the family members about the rules so that they are aware of them and training for informal interpreters and patients is recommended [19]. Accessibility of professional interpreters may increase patient satisfaction and improve medical outcomes, but requires a well-functioning organisation [15, 20]. Fatahi et al. [21] found that interpreters perceived themselves to be members of staff, a view shared by some of the patients—but not the staff themselves. The authors concluded that interpreters ought to be more integrated in the medical care system [21].

To use a professional interpreter is also important for enhancing the patient-care provider relationship and patient centeredness [22]. A patient-centred approach explores the patient's main reason for the visit, concerns, and need for information. It seeks an integrated understanding of the patient's world—that is his/her whole being, emotional needs, and life issues. It enhances the continuing relationship between the patient and the health care provider and helps them to identify what the problem is and to take decisions together [23]. 

The patient is conscious and aware of interactions between the participants during the consultation and the smallest details of how the communication between the participants' works can have a sizeable impact on the eventual outcomes [8]. Our study has indicated that a patient-centred approach is important to support patient self-management, including increased patient participation in discussing and setting goals for treatment, in agreement with Lewin et al. [24] and Kinnersley et al. [25].

Awareness of the patient's cultural views was not deemed important by our participants, as it was in a previous study by Harmsen et al. [26], especially when the patient has more or less adapted socially and psychologically to their new culture. However, in order to achieve a more patient-centred care, there is a need of cultural competence for responding on patients' preferences and goals at the interpersonal level as well as at the health system level [27]. The aim of the European TRICC (“TRaining in Intercultural and bilingual Competencies in health and social Care”) project was to enhance intercultural and bilingual awareness and competencies by developing training courses for health care providers, informal interpreters, and students [28]. 

When introducing a third person to two-person communication, it takes time to establish trust. Sufficient consultation time is necessary to obtain a correct and complete medical history and to adopt a patient-centred approach in which decision making is shared. 

Strength of this study is the opportunity we had to gain insights into the perspectives of all three groups of participants in the consultations by using triangulation and to give a description of these perspectives. We received a richness of data, where the divergence and convergence of the findings, illustrated with quotations from the three views, are a contribution to a deeper understanding of communication problems. We have found a few similar studies in the literature. The validity of the study is good, since the study has been accustomed to the common different stages in a qualitative research interview and the use of interpreters trained in the research field [29]. Since it is a qualitative study, the sampling is “purposeful”; that is, “intentionally selected according to the needs of the study” [30].

A limitation is that there are few patients of three different countries involved in the study. The subjectivity of patients' responses is prone to be influenced by the characteristics of each patient. Nevertheless, each interview is unique and the findings are contextual. Since it is a qualitative study, the findings cannot be generalized, but may be transferred to other contexts with similar characteristics [31]. The language during the interviews might have been influenced by the fact that some of the interpreters and GPs were immigrants themselves. The first author and interviewer is a GP herself, with personal experiences, preconceptions, and expectations which may have influenced the research process [32, 33]. The reliability of the printouts is good, since control interception has been made and the informants had the opportunity to correct them in their own language. Since data analyses were performed by two of the authors independently, it contributes to the reliability of the results.

### 4.2. Conclusion

This paper has highlighted feelings of frustration and insecurity when interpretation and relationships are suboptimal. Strategies for reaching a successful consultation may therefore be needed for all three participants during consultations. To transform the triangular meeting between an immigrant patient, an interpreter, and a GP from an encounter to a real meeting, this study has indicated that there is a need for a professional interpreter, for the GP to use a patient-tailored approach and to have sufficient consultation time. Use of professional interpreters is recommended, as is developing cultural competence. Further research in this field is needed in order to obtain a deeper understanding of the triangular meeting. 

## Figures and Tables

**Table 1:** Characteristics of the patients, interpreters and GPs.

Participants (*n* = 29)	Age	Gender	Country of origin	Length of residency in Sweden	Mother tongue
	<65 yrs old: *n* = 8	Male: *n* = 1	Chile: *n* = 4		Spanish
Patients (*n* = 10)	>65 yrs old: *n* = 2	Female: *n* = 9	Iran: *n* = 3	4–33 years	Persian
			Turkey: *n* = 3		Turkish

	Age	Gender	Country of origin	Length in profession	Mother tongue

	<50 yrs old: *n* = 4	Male: *n* = 2	Syria: *n* = 1		Spanish
	>50 yrs old: *n* = 5	Female: *n* = 7	Iran: *n* = 3	<1–28 years	Persian
Interpreters (*n* = 9)			Turkey: *n* = 2		Turkish
			Uruguay: *n* = 2		
			Sweden: *n* = 1		

	<50 yrs old: *n* = 6	Male: *n* = 3	Iran: *n* = 1		Persian
GPs (*n* = 10)	>50 yrs old: *n* = 4	Female: *n* = 7	Equatorial Guinea: *n* = 1	Many years: *n* = 10	Icelandic
			Iceland: *n* = 1		Swedish
			Sweden: *n* = 7		

**Table 2:** Content analysis: examples of a meaning unit, a condensed meaning unit, a subtheme, and a theme from the content analysis of patient's experiences and reflections pertaining to a consultation.

Meaning unit	Condensed meaning unit	Condensed meaning unit	Subtheme	Theme
	Description similar to the text	Interpretation of the underlying meaning		
*“I want the interpreter to translate or convey exactly how I feel, in other words, my feelings, emotions, and experiences, that is, not only the verbal thing.” *(p10)	The patient says that she wants to have not only an exact verbal translation, but also a translation with feelings, emotions, and experiences.	The patient has a need for interpretation with words, feelings, and experiences.	Need for verbal and emotional interpretation	Professional interpretation

**Table 3:** Participants' different perspectives concerning the themes.

Theme	Patients	Interpreters	GPs
The interpretation process			
Means of interpreting	(i) Establish trust (ii) Translate and convey everything	(i) Translate every word precisely (ii) Neutrality (iii) Not disrupt (iv) Know the technique	(i) Different techniques (ii) Just a voice in the room (iii) Language machines (iv) Empathy
Means of informing	(i) Adjust info to culture and level of knowledge (ii) Everyday language	(i) Tell a little (ii) Adapt to knowledge (iii) Written info	(i) Adjust your way of communication

The meeting itself			
Individual tailored approaches	(i) Kind response (ii) Polite and respectful treatment (iii) *“Care for me” *	(i) Individual approach (ii) Patience (iii) Respect (iv) Patient's needs and wishes (v) Medical authority	(i) Mutual understanding (ii) Individuality (iii) Main reason (iv) Listen (v) Professionalism (vi) Respect (vii) Autonomy (viii) No prejudices
Consultation time	(i) Tell everything (ii) Proper examination (iii) Ask questions	(i) Never sufficient (ii) Frustration (iii) Present his story (iv) Imagination (v) Undivided presence	(i) Need of longer time (ii) Adjust info (iii) Find out what it is all about
The patient's feelings	(i) Frustration not understanding everything	(i) Stress (ii) Uncertainty (iii) *“Must give space” *	(i) *“Talking past each other” * (ii) *“Feels offended”* (iii) Expect professional IP without judgement
The role of family members	(i) Give security (ii) Support (iii) Interpret	(i) Interfere (ii) Incorrect interpretation (iii) Incomplete info (iv) Cause dilemmas	(i) Not to divulge confidential info (ii) Create confidence

**Table 4:** Effects of the interpretation process and the quality of the patient-GP relationship on the success of a consultation.

(1)		
Good interpretation (P + IP)?	Yes*	Successful
Good patient-GP relationship (P + GP)?	Yes**	consultation

(2)		
Good interpretation (P + IP)?	No***	Less successful
Good patient-GP relationship (P + GP)?	Yes	consultation

(3)		
Good interpretation (P + IP)?	Yes	Less successful
Good patient-GP relationship (P + GP)?	No****	consultation

(4)		
Good interpretation (P + IP)?	No*****	Unsuccessful
Good patient-GP relationship (P + GP)?	No******	consultation

A successful consultation embraces good interpretation *and* a good meeting between patient and GP and may therefore be defined as a real meeting and not just an encounter.

*GP10: “A good interpreter who has extensive experience translates quickly; uses shorter sentences, not very long explanations … without the medical content being compromised.”

**IP7: “… the doctor's trust towards his patients and patients' confidence in their doctor  … it requires a great deal of patience on both sides….”

***P4: “It has not gone well the times we have had an interpreter. The interpreter could not translate into Swedish.”

****P4: “If I see that the doctor does not understand, then I say that I can see that you do not understand…in that case I have to go to another doctor….”

*****IP7: “…To give a fast interpretation and perhaps over-interpret…due to be flexibility… a tendency to make what patient says better or to over-interpret it….”

******GP5: “… but is it the case when the interpretation is not working you lose the touch….”

## Appendix

### A. Interview Guide at Semistructured Interviews in the Research Project “Health Care on Equal Terms for Immigrants in Sweden”

*Patient*
Could you tell us about your experiences about visiting a GP in primary health care with an interpreter?
(Please give examples. Have you been understood? Have you been able to say what you wanted? Have you received help? Were there any problems, and if so, have they been solved?)
This is hopefully followed by a story where the interview guide will serve as a background and as a memory list to check that relevant facts and experiences are included in the story.
*Interpreter*
Could you tell us about your experiences to interpret immigrant patients from Chile, Turkey and Iran as they are visiting a GP in primary health care? (Please give examples. Do you think the GP understood the patients' problems and the cultural background? Has the patient been helped? Were there any problems, and if so, have they been solved?)
This is hopefully followed by a story where the interview guide will serve as a background and as a memory list to check that relevant facts and experiences are included in the story.
*GP*
Could you tell us about your experiences to have immigrant patients from Chile, Turkey, and Iran on medical consultations and cooperate with the interpreter? (Please give examples. Do you think the patient felt that he/she has been understood and helped? Do you know the patient's cultural background and its influence? Were there any problems, and if so, have they been solved?)
This is hopefully followed by a story where the interview guide will serve as a background and as a memory list to check that relevant facts and experiences are included in the story.
